# Clinical spectrum and long-term outcomes of antibody-negative severe autoimmune encephalitis: a retrospective study

**DOI:** 10.3389/fimmu.2025.1591771

**Published:** 2025-05-15

**Authors:** Fangfang Li, Yu He, Xiaoqian Chen, Ali Yang, Jiewen Zhang, Weizhou Zang

**Affiliations:** ^1^ Department of Neurology, Henan Provincial People’s Hospital, Zhengzhou University People’s Hospital, Zhengzhou, Henan, China; ^2^ Department of Neurology, Henan University People’s Hospital, Zhengzhou, China; ^3^ The Second Affiliated Hospital of Luohe Medical College, Luohe, Henan, China

**Keywords:** antibody-negative, severe, autoimmune encephalitis, clinical characteristics, prognosis

## Abstract

**Objective:**

The aims of the study were to characterize the clinical manifestations and outcomes of patients with antibody-negative severe autoimmune encephalitis (AE).

**Methods:**

This retrospective, monocentric study recruited patients from the Neurology Department of Henan Provincial People’s Hospital between April 2017 and December 2023. All patients underwent neural antibody testing in both blood and cerebrospinal fluid (CSF) and met the diagnostic criteria for autoantibody-negative but probable severe AE, with available 1-year follow-up data.

**Results:**

In total, 124 patients with autoantibody-negative severe AE were analyzed. Among them, 27.4% achieved good functional outcomes at discharge. Older age (OR 1.034, 95% confidence interval [CI] 1.010-1.058, *p* = 0.004) and the presence of dyskinesia/dystonia (OR 8.463, 95% CI 3.282-21.820, *p* < 0.001) were predictive of poor short-term outcomes. At the 1-year follow-up, 54.8% experienced favorable long-term outcomes. Independent predictors of unfavorable long-term outcomes included older age (OR 1.076, 95% CI 1.018-1.136, *p* = 0.009), longer hospital stays (OR 1.264, 95% CI 1.105-1.446, *p* = 0.001), the presence of refractory status epilepticus (OR 14.765, 95% CI 1.759-123.935, *p* = 0.013) and higher CASE scores at discharge (OR 2.079, 95% CI 1.450-2.980, *p* < 0.001). Additionally, 30.6% of patients had relapsed, with refractory status epilepticus being an independent risk factor for relapse.

**Conclusion:**

Although patients with antibody-negative severe AE experience significant disability in the early stages of their disease, the majority eventually regain independent functioning. Older age at disease onset, longer hospital stays, the presence of refractory status epilepticus and higher CASE scores at discharge may predict a poor long-term prognosis.

## 1 Introduction

Autoimmune encephalitis (AE) is an immune-mediated disease which mainly affects the central nervous system, often manifesting with different combinations of subacute or acute cognitive decline, new-onset seizures, speech dysfunction, sleep disorders, movement disorders, autonomic nervous system dysfunction, disturbance of consciousness, and central hypoventilation (1).

The pathogenesis of AE involves multiple factors, with key processes including environmental triggers, immune activation, and neuronal damage. Environmental factors, such as viral infections, tumor antigens, or drug exposure, activate T cells via antigen-presenting cells. This activation subsequently promotes the differentiation of B cells into plasma cells, leading to the secretion of pathogenic autoantibodies. During this process, pro-inflammatory cytokines enhance the secretion of antibodies by plasma cells. These antibodies disrupt neurological function through two primary mechanisms (1) directly inhibiting the physiological activity of synaptic receptors (e.g., NMDA receptors) (2) inducing excessive neuronal excitation and subsequent neuronal injury. Additionally, inflammatory responses compromise the integrity of the blood-brain barrier, enabling antibodies to infiltrate the central nervous system and bind to neuronal surface antigens, thereby disrupting and damaging neuronal function. Beyond humoral immunity, other critical pathological mechanisms include T cell-mediated cytotoxicity, microglial activation, and focal inflammatory infiltration. While the clinical features of antibody-positive AE are relatively well-defined, the antibody-negative subtype, which constitutes more than half of all cases, lacks specific antibody markers. Its heterogeneous clinical manifestations and complex pathogenesis, potentially involving unknown antigens or T cell-mediated damage, necessitate further research into biomarkers and precision medicine approaches. Moreover, while changes in antibody titer and status can guide subsequent immunotherapy for antibody-positive AEs, there are limited references for antibody-negative AE patients (3, 4).

To address these gaps, Francesc et al (5) proposed diagnostic criteria for autoantibody-negative AE in 2016. However, owing to the unique disease mechanisms, clinical characteristics, and prognosis, there are still many limitations in our understanding of antibody-negative AE. Recent published studies have explored features and long-term outcomes of antibody-negative AE, including pediatric AE cases (6–8). Nevertheless, comprehensive studies focusing on severe antibody-negative AE are scarce. Besides, AE patients may develop severe and disabling encephalitis due to impaired consciousness, refractory seizures, or respiratory failure. Reports indicate that approximately 40% of patients who have antibody-negative AE require ICU treatment, and 12% to 40% will die because of AE (9, 10). Therefore, it is crucial for clinicians to promptly recognize these AEs, understand their disease trajectories and implement effective interventions to mitigate the devastating outcomes and promote neurological function recovery (11).

In the present study, we aimed to describe the clinical characteristics of patients diagnosed with antibody-negative AEs, as well as to identify potential factors influencing their short-term outcomes upon hospital discharge and long-term outcomes.

## 2 Methods

### 2.1 Study population

In the present study, we retrospectively identified a consecutive series of patients admitted to the Neurology Department of Henan Provincial People’s Hospital between April 2017 and December 2023. Our study was approved by the Ethics Committee of Henan Provincial People’s Hospital, with the approval number being Ethics Review No. 129 (granted in 2022). Written informed consent was obtained from the immediate family members of the patients. Severe AE refers to cases that fulfill the diagnostic criteria for AE and necessitate admission to the NCU for monitoring and therapeutic intervention. Inclusion criteria were as follows, (1) fulfilled the diagnostic criteria for possible AE (5): subacute onset of abnormalities in memory, mental status, or psychiatric symptoms, accompanied by at least one of the following new focal central nervous system(CNS) findings: unexplained seizures, CSF pleocytosis, or MRI features suggestive of encephalitis, after reasonable exclusion of other potential etiologies; (2) fulfilled the diagnostic criteria for autoantibody-negative but probable AE (5): rapid progression in abnormalities of memory, mental status, or psychiatric symptoms, which cannot be attributed to well-defined syndromes of AE, is accompanied by the absence of well-characterized autoantibodies in both serum and cerebrospinal fluid (CSF), as well as the presence of at least two of the following indicators: MRI abnormalities indicative of AE, AE-related CSF changes (such as pleocytosis, CSF-specific oligoclonal bands, or an elevated IgG index), or a brain biopsy demonstrating inflammatory infiltrates, and other potential etiologies have been reasonably excluded; (3) no autoimmune antibody was detected in both the serum and CSF; (4) Upon admission, given the critical condition of the patient, ICU admission was necessary for intensive monitoring and therapeutic intervention. The mRS score ranged from 4 to 5; (5) clinical evaluations at admission, discharge and 1 year after AE attack were performed and recorded; (6) with complete medical records. The exclusion criteria were: (1) patients with well-characterized autoantibodies in serum and/or CSF; (2) patients with infectious encephalitis; (3) patients diagnosed with toxic encephalopathy, metabolic encephalopathy, or brain tumors prior to the AE; (4) patients diagnosed with definite limbic encephalitis, acute disseminated encephalomyelitis, Bickerstaff brainstem encephalitis, or Hashimoto’s encephalopathy; (5) patients with mRS scores of 0–3 upon admission; (6) patients with follow-up periods less than 1 year or refused to follow-up; (7) patients with incomplete medical records.

### 2.2 Data collection

Patient’s data were obtained from medical and hospitalization records at baseline and subsequently, including (1) demographic characteristics (age and sex at baseline) (2); clinical symptoms of epilepsy, working memory deficit, altered mental status, psychiatric symptoms, and focal CNS signs (language problem, dyskinesia/dystonia, ataxia, brainstem dysfunction, and weakness) (3); laboratory indicators, including WBC (white blood cell), NLR (Neutrophil-to-lymphocyte ratio), CRP (C-reactive protein), and Qalb (CSF albumin/Serum albumin, reflecting blood-brain barrier disruption) at baseline. Refractory status epilepticus was defined as the persistence of status epilepticus despite prescribing and administering at least two intravenous anti-seizure medications at appropriate doses. Modified Rankin Scale (mRS) and Clinical Assessment Scale in Autoimmune Encephalitis (CASE) score (12) were assessed at admission, at discharge and 1year after discharge. Blood tests including measurements of WBC count, neutrophils, lymphocytes, and CRP levels were conducted on all patients within 24 hours after admission and prior to immunotherapy. All patients underwent MRI scans as well as CSF studies. CSF was considered pleocytosis if there was an elevation in WBC (> 8/mm^3^). When multiple lumbar punctures were conducted, CSF pleocytosis, oligoclonal bands, IgG index and Qalb were recorded from the sample yielding the first result. The immunotherapy regimen includes both first-line and second-line therapies. The first-line therapy consists of large doses of glucocorticoids, intravenous immunoglobulin, or plasma exchange, while the second-line therapy comprises rituximab, cyclophosphamide, and tocilizumab. Combined first-line therapy was defined as the concurrent or sequential use of two or more first-line therapies. Clinical response was defined as mRS improvement ≥1 point after initial treatment (13). Relapse was defined as clinical exacerbation occurring at least 2 months after the onset of the most recent episode, and which is not attributable to the immunosuppressive therapy regimen or other pathogenesis. Poor neurological outcome was defined as an mRS score >2 at 1 year post-discharge.

### 2.3 Antibody test

Both serum and CSF samples from all patients were evaluated for anti-N-methyl-D-aspartate receptor (NMDAR) antibody, anti-contactin-associated protein-like 2 (CASPR2) antibody, anti-α-amino-3-hydroxy-5-methyl-4-isoxazolepropionic acid receptor 1 (AMPA1R) antibody, anti-AMPA2R antibody, anti-γ-aminobutyric acid-B receptor (GABABR) antibody, and anti-leucine-rich glioma-inactivated 1 (LGI1) antibody, using indirect immunofluorescence (IIF) or cell-based assay (CBA). Additionally, with the emergence of new antibodies, patients included in recent years have also received evaluations for anti-dipeptidyl-peptidase-like protein-6 (DPPX), anti-mGluR5, glycine receptor 1 (GlyR1), anti-glutamic acid decarboxylase 65 (GAD65), IgLON family member 5 (IgLON5), and anti-Dopamine 2 receptor (D2R). All patients were also evaluated for AQP4, MOG, GFAP, and MBP to exclude known inflammatory demyelinating diseases. All patients were evaluated for Amphiphysin, CV2, Ma2, Ri, Yo, and Hu to exclude paraneoplastic syndrome. Antibody negativity was defined as autoimmune antibodies not being detected in either serum or CSF tests.

### 2.4 Statistical analysis

The data were processed using SPSS 21.0 software (IBM Corp.). Continuous variables, which were non-normally distributed, were expressed as medians with inter-quartile ranges (IQR) and analyzed using the Mann-Whitney U test. The Chi-Squared test was utilized to compare the differences of categorical variables. The Receiver Operating Characteristic (ROC) curve was applied to assess the predictive ability of CASE at admission for predicting short-term and long-term outcomes in antibody-negative severe AE patients. Finally, univariate analysis was first conducted with a significance level of *p* < 0.1 to screen for factors related to short-term and long-term outcomes, as well as death. Subsequently, multivariate logistic stepwise regression analyses (forward: LR) were performed to further confirm and identify risk factors associated with these outcomes. p<0.05 was defined as statistically significant.

### 2.5 Ethics statement

The study was approved by the Ethics Committee of Henan Provincial People’s Hospital and was conducted in compliance with local legislation and institutional requirements. Written informed consent for participation in this study was obtained from the participants’ legal guardians.

## 3 Results

### 3.1 Demographic and clinical characteristics

A total of 124 patients, eligible based on inclusion and exclusion criteria, and diagnosed with antibody-negative severe AE, were enrolled in this study. The patients had a median age of 49 years (28.5–59), with a male predominance of 80 (64.5%). The median lengths of hospital stay and ICU stay were 20 days (14–30) and 8 days (5–13), respectively. Baseline characteristics of patients are presented in **Table 1**. The most frequent clinical features included language problems (98.4%), brain stem dysfunction (92.7%), impaired consciousness (88.7%), psychiatric symptoms (78.2%), and memory dysfunction (77.4%). Of those, 83.9% (104 patients) were admitted to the ICU due to central hypoventilation. Additionally, the median CASE score upon admission was 23 (18.25-25), and the median mRS score upon admission was 5 (4.25-5). Immunotherapy was initiated at 4 days (range: 2-9) from the onset. Of the patients, 72.6% received combined first-line therapy, while 20.2% received second-line therapy. In total, 75.0% of patients responded clinically, and the median CASE score at discharge was 9 (6–18). At the 1-year follow-up, 68 patients (54.8%) achieved a good clinical outcome, with a median CASE score of 4 (2-11.5) and a median mRS score of 2 (1–4).

**Table 1:** Baseline characteristics based on 1 year outcome.

Variables	All (n=124)	Favorable outcome (n=68)	Unfavorable outcome (n=56)	*p* value
Age, year; median, IQR	49 (28.5-59)	37.5 (18.3-54.8)	57 (39.3-66)	0.000
Male, n (%)	80 (64.5)	44 (64.7)	36 (64.3)	0.961
Hospital stays, days; median, IQR	20 (14-30)	18.0 (13.3-23.8)	24.5 (15.0-39.8)	0.003
ICU stays, days; median, IQR	8 (5-13)	6.5 (4-9)	10.5 (6-19.8)	0.000
Clinical features, n (%)				
Refractory status epilepsy	40 (32.3)	16 (23.5)	24 (42.9)	0.022
Memory dysfunction	96 (77.4)	45 (66.2)	51 (91.1)	0.001
Psychiatric symptoms	97 (78.2)	45 (66.2)	52(92.9)	0.000
Disturbance of consciousness	110 (88.7)	56 (82.4)	54 (96.4)	0.014
Language problem	122 (98.4)	66 (97.1)	56 (100)	0.501
Dyskinesia/dystonia	90 (72.6)	42 (61.8)	48 (85.7)	0.003
Gait instability and ataxia	92 (74.2)	43 (63.2)	49 (87.5)	0.002
Brain stem dysfunction	115 (92.7)	59 (86.8)	56 (100)	0.004
Weakness	86 (69.4)	35 (51.5)	51 (91.1)	0.000
Hypoventilation	104(83.9)	50(73.5)	54(96.4)	0.001
CASE at admission; median, IQR	23 (18.25-25)	20.5 (16.3-24.0)	24.5 (23.0-26.0)	0.000
mRS at admission; median, IQR	5 (4.25-5)	5 (4-5)	5 (5-5)	0.000
WBC at admission; median, IQR	9.39 (7.31-12.87)	9.44 (7.57-12.75)	7.27 (7.17-13.39)	0.998
NLR at admission; median, IQR	6.75 (3.23-11.48)	5.60 (2.85-10.65)	8.45 (4.73-13.24)	0.026
CRP at admission; median, IQR	17.21 (1.82-52.33)	7.52 (0.58-33.12)	28.73 (3.49-90.44)	0.005
Qalb at admission; median, IQR	13.6 (9.19-21.81)	12.66 (7.83-21.99)	14.01 (9.98-21.61)	0.277
Treatment profiles				
Onset to immunotherapy (days)	4 (2-9)	3 (1-8)	5 (2-10.75)	0.155
Combined first-line therapy, n (%)	90 (72.6)	46 (67.6)	44 (78.6)	0.175
Second line therapy, n (%)	25 (20.2)	12 (17.6)	13 (23.2)	0.442
Outcome				
Clinical respond, n (%)	93 (75.0%)	67 (98.5)	25(44.6)	0.000
CASE at discharge; median, IQR	9 (6-18)	6.5 (6.0-8.0)	19.0 (10.3-24)	0.000
CASE at 1 year; median, IQR	4 (2-11.5)	2 (2-3)	13.5 (7.0-25.5)	0.000
mRS at 1 year; median, IQR	2 (1-4)	2 (1-2)	4.0 (3.3-6.0)	0.000
Relapse (%)	38 (30.6)	17 (25.0)	21 (37.5)	0.133
Death (%)	18 (14.5)	0 (0.00)	18 (32.1)	0.000

IQR, interquartile range; WBC, white blood cell; NLR, neutrophil to lymphocyte ratio; CRP, C-reactive protein; Qalb, albumin ratio.

Compared with patients who had good functional status at the 1-year follow-up, the clinical variables associated with unfavorable outcomes included older age, longer hospital stays, and longer ICU stays. Besides, they were more likely to exhibit a higher incidence of refractory epilepsy, memory dysfunction, psychiatric symptoms, impaired consciousness, dyskinesia/dystonia, gait instability, brainstem dysfunction and weakness. Moreover, patients with unfavorable outcomes at the 1-year follow-up had significantly higher admission scores for CASE and mRS, as well as NLR and CRP levels. Also, we found that patients with unfavorable outcomes at 1-year follow-up showed limited clinical response to immunotherapy, had significantly higher CASE scores at discharge, and experienced a higher incidence of death during follow-up compared to those with favorable outcomes.

### 3.2 Factors associated with poor short-term functional outcome

At discharge, 34 patients (27.4%) achieved good functional outcomes. We performed univariate analysis to identify predictors of poor prognosis. In univariate analysis, older age; longer hospital stays; longer ICU stays; higher CASE scores and mRS upon admission; as well as higher CRP levels upon admission were associated with poor short-term functional outcomes. Additionally, patients with poor short-term functional outcomes exhibited a higher incidence of psychiatric symptoms, memory deficits, impaired consciousness, dyskinesia/dystonia, ataxia, brainstem dysfunction, weakness and hypoventilation. In subsequent multivariate logistic regression analyses, older age (OR 1.034, 95%CI 1.010-1.058, *p* = 0.004) and presence of dyskinesia/dystonia (OR 8.463, 95% CI 3.282-21.820, *p <*0.001) were significantly associated with poor short-term functional outcomes (**Table 2**). The results suggest that for every 1-year increase in patient age, the odds of a poor prognosis increase by a factor of 1.034. Furthermore, the risk of poor prognosis in patients with dyskinesia/dystonia was 8.463 times the risk in patients without dyskinesia/dystonia (**Table 2**).

**Table 2:** Multivariate Logistic regression analysis for the factors associated with short term poor clinical outcome.

Variables	OR	95%CI	*p* value
Age of onset	1.034	1.010-1.058	0.004
Dyskinesia/dystonia	8.463	3.282-21.820	0.000

### 3.3 Factors associated with poor long-term functional outcome

Univariate analyses and subsequent multivariate binary logistic regression analyses were further conducted to identify variables associated with poor long-term outcomes. In univariate analysis, patients with poor long-term outcomes tended to be older, have longer hospital stays and ICU stays, higher CASE scores and mRS upon admission, elevated CRP levels at nadir, and higher CASE scores at discharge. Additionally, they had a higher proportion of psychiatric symptoms, memory deficits, impaired consciousness, dyskinesia/dystonia, ataxia, brainstem dysfunction, weakness, and refractory status epilepsy (RSE), as well as a lower likelihood of clinical response. However, after multivariate adjustment, we found that age of onset (OR 1.076, 95% CI 1.018-1.136, *p* =0.009), hospital stays (OR 1.264, 95% CI 1.105-1.446, *p* =0.001), refractory status epilepticus (OR 14.765, 95% CI 1.759-123.935, *p* =0.013), and CASE scores at discharge (OR 2.079, 95% CI 1.450-2.980, *p* < 0.001) were independently associated with unfavorable long-term outcomes (**Table 3**).

**Table 3:** Multivariate Logistic regression analysis of factors associated with 1-year poor clinical outcome.

Variables	OR	95%CI	*p* value
Age of onset	1.076	1.018-1.136	0.009
Refractory status epilepticus	14.765	1.759-123.935	0.013
Hospital stays	1.264	1.105-1.446	0.001
CASE at discharge	2.079	1.450-2.980	0.000

**Figure 1:**
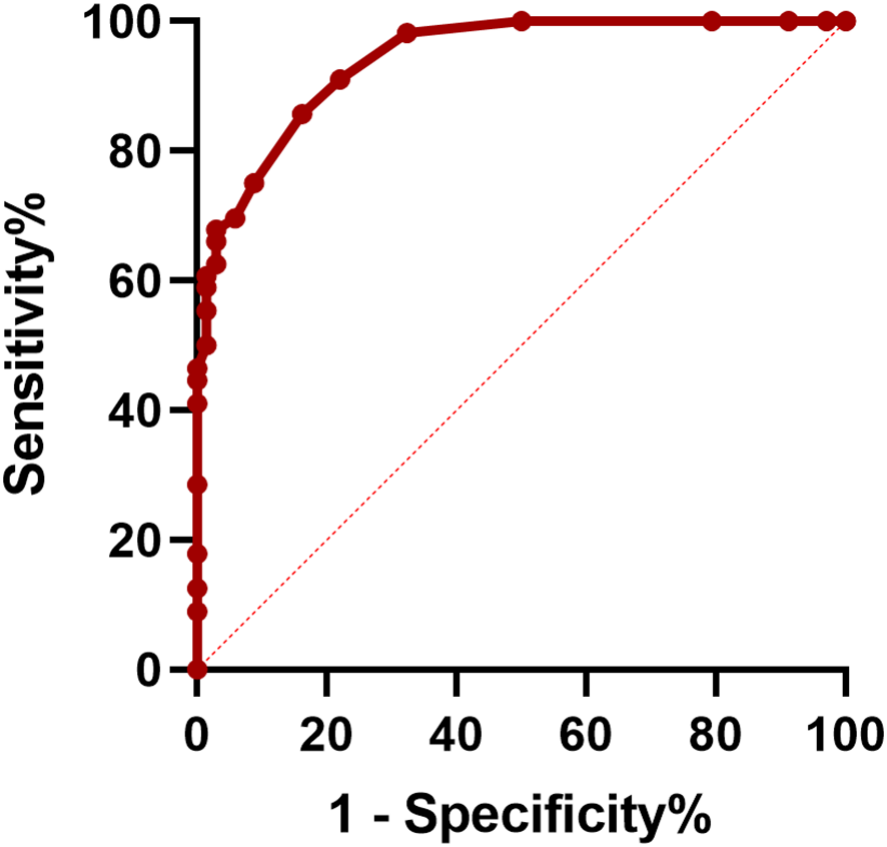
Receiver operating characteristic (ROC) curve of CASE at discharge in predicting 1-year poor clinical outcome.

Furthermore, ROC curves were employed to assess the predictive value of CASE scores at discharge for long-term outcomes and to determine the optimal cut-off value. The results showed that the area under the curve (AUC) of the prediction probability was 0.941 (95% CI: 0.905-0.977; *p* < 0.001). The optimal cut-off value of CASE scores at discharge was 9.5 points, with a specificity of 0.838 and a sensitivity of 0.857 (**Figure 1**).

### 3.4 Factors associated with relapse

At 1 year follow-up, 38 patients (30.6%) experienced relapse. Next, we explored factors that may predict relapse in their first year after disease onset. The variables entered into the multivariate model included age at onset, hospital stay duration and presentations of refractory epilepsy. Multivariate logistic regression analysis indicated that only presentation of refractory status epilepsy (OR 3.667, 95% CI 1.631-8.241, *p* =0.002) was independent risk factor for relapse (**Table 4**).

**Table 4:** Predictors of relapse by multivariate analysis.

Variables	OR	95%CI	*P*-value
Refractory status epilepticus	3.667	1.631-8.241	0.002

### 3.5 Differences between survivors and non-survivors

**Figure 2:**
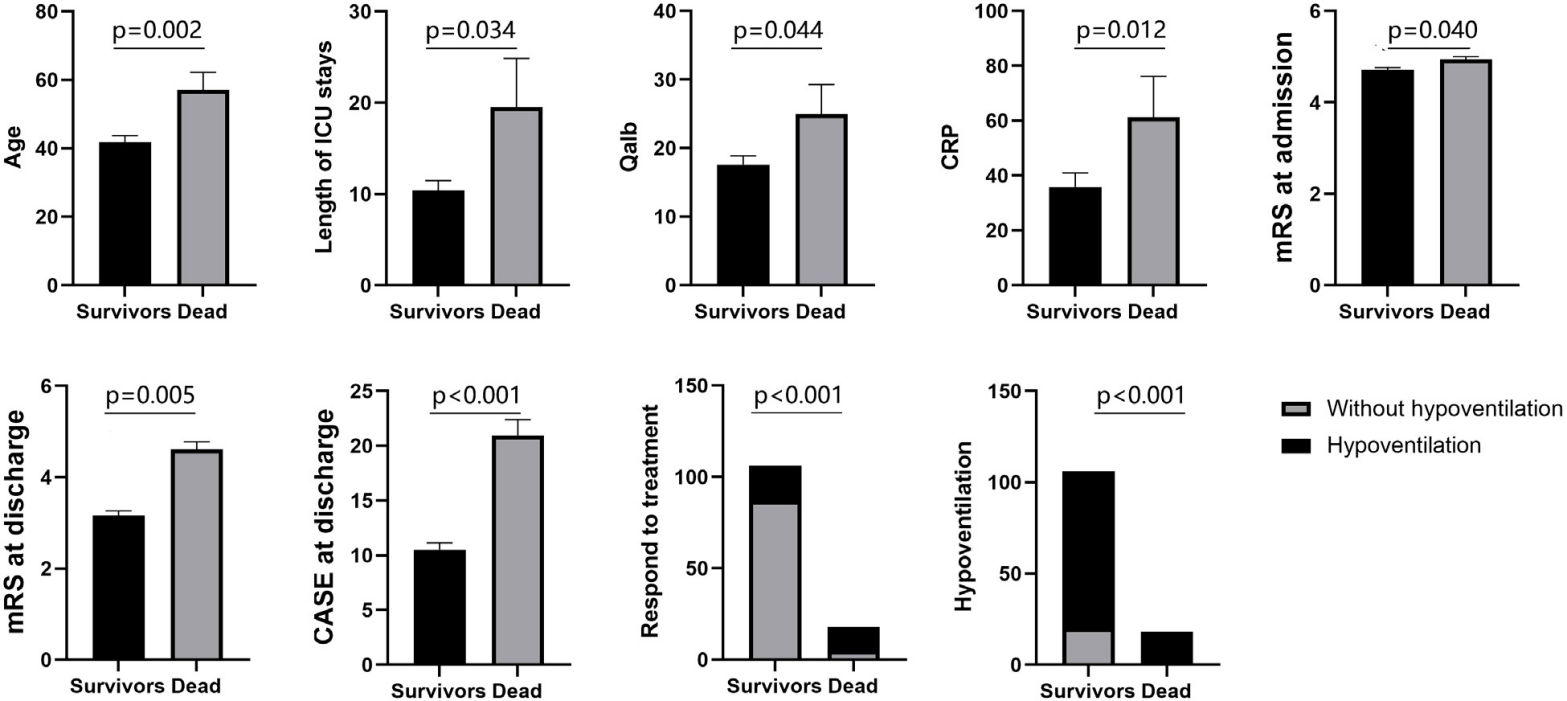
Comparisons of patients between survivors and dead.

At the 1-year follow-up, 18 patients (14.5%) had died. We then compared the characteristics of the surviving patients with those of the deceased. Among the surviving patients, 86(81.1%) patients required assisted ventilation due to hypoventilation. Among the deceased patients, 18(100%) patients needed assisted ventilation. On average, the non-survivors in this study were older, had longer ICU stays, exhibited higher levels of Qalb and CRP, and had higher CASE scores and mRS at discharge. Moreover, they had a lower likelihood of responding to immunotherapy (**Figure 2**).

## 4 Discussion

In this study, we retrospectively analyzed the clinical data of 124 antibody-negative severe AE patients and found: First, one year after disease onset, the frequency of favorable functional outcomes was 54.8%, the frequency of relapse was 30.6%, and the frequency of death was 14.5%. Second, older age and the presence of dyskinesia/dystonia was positively associated with poor short-term functional outcomes. Third, older age, longer hospital stays, the presence of refractory status epilepticus, and higher CASE scores at discharge were independently associated with unfavorable long-term outcomes. Fourth, the presentation of refractory status epilepsy indicated an increased possibility of relapse. Fifth, deceased patients were older at onset, had severe clinical manifestations, and showed a pronounced inflammatory state. These findings may enhance neurologists’ and intensivists’ knowledge of valuable clinical characteristics and information related to prognosis.

The frequency of unfavorable 1-year outcomes was similar to that of favorable outcomes and comparable to previous reports (14, 15). Notably, approximately half of patients were not able to function independently within one year after disease onset, possibly because antibody-negative AE is a highly heterogeneous syndrome with disparate pathological mechanisms that may be refractory to conventional immunotherapy (14, 16). Besides, 27.4% of patients attained favorable outcomes at discharge, and 45.2% of patients attained favorable outcomes at the last follow-up, consistent with prior literature demonstrating that a significant proportion of patients continue to improve within one year after disease onset (17). This underscores the crucial importance of early diagnosis, timely treatment, and long-term management throughout the acute, subacute, and chronic phases for the outcomes of antibody-negative severe AE.

Our study found that advanced age was consistently associated with unfavorable short-term and long-term outcomes. This can be attributed to decreased brain functional reserves, reduced tolerance to immunotherapy, and an increased risk of medical complications. Furthermore, age-associated impaired CNS lymphatic drainage and abnormal microglia activation may further exacerbate neuroinflammation (18, 19). Notably, dyskinesia/dystonia was found independently associated with unfavorable short-term outcomes. The association between dystonia and AE has been widely reported and dyskinesia/dystonia has been described as an independent predictor of poor functional outcomes six months post-onset (20). However, the underlying pathomechanisms remain incompletely understood. A possible explanation is that the abnormal immune response leads to dysfunctional transmission and secretion of dopamine and GABA, as well as the death of dopaminergic neurons in the nigrostriatal pathway, ventral brainstem, and spinal cord, thereby interfering with the function of the basal ganglia circuit (21). As timely treatment can effectively mitigate movement disorders in most patients (22, 23), early diagnosis and aggressive therapy are essential and may contribute to improved short-term outcomes, according to our study.

Epilepsy exhibits a significantly higher incidence in antibody-negative AE, ranging from approximately 42.4% to 81.28% (24). Furthermore, the incidence of RSE in this context has been documented to be between 7% and 29.9% (7, 14, 17). In the present study, the frequency of RSE was 32.3%. The reason for this relatively higher frequency in our study may be that the included patients had more severe cases, with a median CASE at its nadir being 23. Besides, our analysis also showed a positive correlation between the occurrence of RSE and both the risk of relapse and long-term outcomes.

As the most common initial symptom of AE, RSE represents a highly intractable and life-threatening condition that frequently leads to admission to the ICU and carries a poor prognosis. The potential pathogenesis may be related to cytotoxic T cells inducing neural death and gliosis, elevated levels of pro-convulsant cytokines, as well as autoimmunity associated structural brain abnormalities (25, 26). However, the exact pathogenesis remains intricate due to undetermined etiologies or, alternatively, because some cases may represent undiagnosed antibody-positive conditions with rarer specificities that are not detected by existing diagnostic kits, necessitating further exploration.

CASE has been developed recently as a supplement to the mRS, which has limitations in that it solely concentrates on motor function (12). CASE offers a more comprehensive evaluation of disease severity in patients with AE by quantifying various symptoms, and it has been reported to be associated with early ICU admission, outcomes, and relapse of AE (27–29). In the present study, we found that higher CASE scores upon discharge were independently associated with adverse long-term outcomes. A CASE score exceeding 9.5 indicated that patients may not acquire functional independence within the following year, offering clinicians invaluable insights into prognosis and serves as a reminder to consider more aggressive treatment options as well as maintain closer follow-up.

Additionally, the proportion of patients with central hypoventilation in our study was higher than that reported in other studies (20, 30, 31), and the decreased patients had a higher proportion of hypoventilation. This may be attributed to the more severe conditions of the patients included in our analysis. Upon diagnosis of an AE, first-line therapy, comprising intravenous corticosteroids, intravenous immunoglobulin, or plasma exchange, should be prescribed promptly (32). In the present study, all patients received first-line therapy, and 72.6% of patients underwent combined first-line therapy, reflecting the refractoriness of the disease. However, there was no significant difference in prognosis one year later between patients who received combined first-line therapy and those who did not. Nevertheless, we cannot conclude that combined first-line therapy is futile. The delayed effect of corticosteroids and immunoglobulin may explain this situation. On the contrary, due to the severe consequences of antibody-negative AE, treatment decisions should be deliberate and based on the patient’s age, syndrome, comprehensive examination results, and clinical response.

There are several limitations in the present study. First, the study includes patients from earlier years, a period with fewer immunosuppressant options available, which may limit the generalizability of our findings. Second, the study was a retrospective analysis carried out at a solitary institution. The control of confounding factors was inadequate, and the exclusion of patients with missing data or who refused to follow up further exacerbates the statistical bias. Given the rarity of this disease, the present study included a relatively larger sample size of patients with antibody-negative severe AE in an advanced clinical center, which may help address the deficiencies. Third, the number of patients who died in the present study was small, which limits the analysis of factors associated with death. Fourth, due to various factors, including the inclusion of patients from earlier years, concerns about the cost and side effects of second-line immunotherapy drugs, and the impact of the COVID-19 period, some patients may not have received proper immunotherapy, potentially leading to an underestimation of the effect of immunosuppressants. Fifth, Bonferroni correction was not applied in the multivariate Logistic regression analysis. While we incorporated clinically relevant core variables associated with prognosis and reported exact p-values to improve result transparency, the absence of multiple testing correction may still elevate the risk of Type I errors in identifying prognostic factors. Additional studies are warranted to overcome these shortcomings.

## 5 Conclusion

Our research provides an exhaustive portrayal of the characteristics and prognosis of patients with antibody-negative severe AE. Despite significant disability at the initial stages of the disease, over half of these patients could achieve functional independence within the first year after onset, highlighting the importance of active intervention. Nonetheless, the incidence of relapse and death remains a concern, underscoring the critical need for long-term disease management. In addition, advanced age is an unmodifiable risk factor for all unfavorable clinical outcomes, and refractory status epilepsy is associated with both unfavorable long-term outcomes and relapses, indicating that particular attention should be paid when confronted with these conditions. Moreover, CASE scores upon discharge may assist in predicting long-term prognosis, and deceased patients exhibited a more severe morbid state at their nadir, which should garner clinicians’ high attention. However, further prospective, multicenter clinical trials with a larger sample size are needed to explore unsolved problems and provide the complete picture of antibody-negative severe AE.

## Data Availability

The raw data supporting the conclusions of this article will be made available by the authors, without undue reservation.
