# Supplementary material for: Clinical spectrum and long-term outcomes of antibody-negative severe autoimmune encephalitis: a retrospective study
Source: Front Immunol. 2025 May 15;16:1591771. doi: 10.3389/fimmu.2025.1591771 (PMC12119582; doi:10.3389/fimmu.2025.1591771)
Supplement: Supplementary file 1 [file Table1.docx]

Supplementary Material

# Supplementary Tables

Supplementary Table 1. Univariate logistic regression analysis for the factors associated with short term poor clinical outcome.

| Variables | OR | 95%CI | *p* value |
| --- | --- | --- | --- |
| Age of onset | 1.030 | 1.010-1.051 | 0.004 |
| Hospital stays | 1.040 | 1.002-1.080 | 0.040 |
| ICU stays | 1.071 | 1.002-1.145 | 0.042 |
| CASE score at admission | 1.232 | 1.114-1.364 | 0.000 |
| mRS score at admission | 6.663 | 2.726-16.288 | 0.000 |
| CRP | 1.009 | 0.999-1.019 | 0.075 |
| Psychiatric symptoms | 4.146 | 1.684-10.208 | 0.002 |
| Memory dysfunction | 4.676 | 1.908-11.461 | 0.001 |
| Consciousness | 3.074 | 0.989-9.555 | 0.052 |
| Dyskinesia/dystonia | 7.755 | 3.186-18.877 | 0.000 |
| ataxia | 4.111 | 1.734-9.749 | 0.001 |
| Brainstem dysfunction | 11.407 | 2.236-58.196 | 0.003 |
| Weakness | 3.937 | 1.705-9.093 | 0.001 |

Supplementary Table 2. Univariate logistic regression analysis of factors associated with 1-year poor clinical outcome.

| Variables | OR | 95%CI | *p* value |
| --- | --- | --- | --- |
| Age of onset | 1.030 | 1.018-1.059 | 0.000 |
| Hospital stays | 1.060 | 1.025-1.097 | 0.001 |
| ICU stays | 1.118 | 1.050-1.190 | 0.001 |
| CASE score at admission | 1.357 | 1.196-1.539 | 0.000 |
| mRS score at admission | 8.561 | 2.774-26.423 | 0.000 |
| CASE score at discharge | 1.590 | 1.316-1.921 | 0.000 |
| CRP | 1.008 | 1.001-1.015 | 0.026 |
| Refractory status epilepticus | 2.437 | 1.128-5.268 | 0.023 |
| Psychiatric symptoms | 6.644 | 2.137-20.657 | 0.001 |
| Memory dysfunction | 5.213 | 1.830-14.852 | 0.002 |
| Consciousness | 5.786 | 1.237-27.067 | 0.026 |
| Dyskinesia/dystonia | 3.714 | 1.519-9.082 | 0.004 |
| ataxia | 4.070 | 1.601-10.344 | 0.003 |
| Hypoventilation | 9.720 | 2.146-44.029 | 0.003 |
| Weakness | 9.617 | 3.419-27.054 | 0.000 |
| Clinical respond | 0.012 | 0.002-0.093 | 0.000 |

Supplementary Table 3. Predictors of relapse by univariate analysis.

| Variables | OR | 95%CI | *P*-value |
| --- | --- | --- | --- |
| age at onset | 0.982 | 0.963-1.000 | 0.054 |
| hospital stays | 1.022 | 0.999-1.046 | 0.067 |
| Refractory status epilepticus | 3.667 | 1.631-8.241 | 0.002 |
